# Neurodynamics of executive control processes in bilinguals: evidence from ERP and source reconstruction analyses

**DOI:** 10.3389/fpsyg.2015.00821

**Published:** 2015-06-15

**Authors:** Karin Heidlmayr, Barbara Hemforth, Sylvain Moutier, Frédéric Isel

**Affiliations:** ^1^Laboratory Vision Action Cognition – EA 7326, Institute of Psychology, Paris Descartes University – Sorbonne Paris Cité, ParisFrance; ^2^CNRS, Laboratory of Formal Linguistics, UMR 7110, Paris Diderot University – Sorbonne Paris Cité, ParisFrance; ^3^Laboratoire de Psychopathologie et Processus de Santé – EA 4057, Institute of Psychology, Paris Descartes University – Sorbonne Paris Cité, ParisFrance; ^4^CNRS, Laboratory of Phonetics and Phonology, Université Sorbonne Nouvelle – Sorbonne Paris Cité, ParisFrance

**Keywords:** executive control, bilingualism, Stroop interference, negative priming, N200, N400, ACC, PFC

## Abstract

The present study was designed to examine the impact of bilingualism on the neuronal activity in different executive control processes namely conflict monitoring, control implementation (i.e., interference suppression and conflict resolution) and overcoming of inhibition. Twenty-two highly proficient but non-balanced successive French–German bilingual adults and 22 monolingual adults performed a combined Stroop/Negative priming task while event-related potential (ERP) were recorded online. The data revealed that the ERP effects were reduced in bilinguals in comparison to monolinguals but only in the Stroop task and limited to the N400 and the sustained fronto-central negative-going potential time windows. This result suggests that bilingualism may impact the process of control implementation rather than the process of conflict monitoring (N200). Critically, our study revealed a differential time course of the involvement of the anterior cingulate cortex (ACC) and the prefrontal cortex (PFC) in conflict processing. While the ACC showed major activation in the early time windows (N200 and N400) but not in the latest time window (late sustained negative-going potential), the PFC became unilaterally active in the left hemisphere in the N400 and the late sustained negative-going potential time windows. Taken together, the present electroencephalography data lend support to a cascading neurophysiological model of executive control processes, in which ACC and PFC may play a determining role.

## Introduction

The bilingual brain can distinguish and control which language is in use. For example, individuals who communicate in more than one language are able to produce words in the selected language and to inhibit the production of words in the non-selected language. This cognitive ability to control multiple languages is assumed to rely on the involvement of different cognitive processes. More generally, cognitive control, also known as executive functions, can be defined as a set of processes involved in managing processes and resources in order to achieve a goal. It is an umbrella term for the neurologically based skills involving mental control and self-regulation. Current psychological and neurobiological theories describe cognitive control either as unitary or as a system fractioned into different sub-processes. Alternatively, hybrid theoretical accounts as proposed by Miyake et al. (2000) attempt to integrate both unifying and diversifying characteristics of executive functions. Miyake et al. (2000) postulate three main executive functions, namely inhibition of dominant responses (“inhibition”), shifting of mental sets (“shifting”) and monitoring and updating of information in working memory (“updating”). In the study presented in this paper, we examined cognitive inhibition but also overcoming inhibition mechanisms.

One of the key discoveries in human cognitive and brain sciences in the past 20 years is the increasing evidence from behavioral, neurophysiological, and neuroimaging studies for the plasticity of executive functions (Dahlin et al., 2008; Li et al., 2014). Psychological research has shown that the efficiency of executive control processes can be influenced among others by multiple language use (for reviews, see Costa et al., 2009; Kroll and Bialystok, 2013; Baum and Titone, 2014; Grant et al., 2014). The rationale for accounting for an improvement of executive control processes in bilinguals is the following: both languages are activated to some degree in bilingual individuals (Van Heuven et al., 1998; Hoshino and Thierry, 2011); therefore, executive control processes are regularly solicited to maintain the target language(s) in a given interactional context and to avoid persistent bidirectional cross-language influences (Blumenfeld and Marian, 2013). This constant training may make these processes more efficient in the long run. A convincing argument in favor of such a bilingualism advantage in executive functioning is empirical evidence of shorter color naming times in conflicting trials of a Stroop task (i.e., incongruency between the word of the color and the ink) in bi- than in monolinguals (Bialystok et al., 2008; Heidlmayr et al., 2014). It is however important to note that although a growing number of behavioral studies investigating control processes in bilingualism show that bilinguals perform better in many executive functions tasks (Kovacs and Mehler, 2009; Prior and Macwhinney, 2009; Gathercole et al., 2010; Hernández et al., 2010; Isel et al., 2012; Kroll and Bialystok, 2013; Kuipers and Thierry, 2013; Marzecová et al., 2013; Heidlmayr et al., 2014), a significant number of studies failed to report such an advantage of bilingualism (Morton and Harper, 2007; Paap and Greenberg, 2013; Antón et al., 2014; Duñabeitia et al., 2014; Gathercole et al., 2014; for reviews, see Costa et al., 2009; Hilchey and Klein, 2011; Kroll and Bialystok, 2013; Valian, 2015). For example, in a large sample of 252 bilingual children (age 10.5 ± 1.8 years), using both a Classic Stroop task (linguistic component) and a Numerical Stroop task (no linguistic component) to disentangle the effects that are due to language processing and those due to control processes, Duñabeitia et al. (2014) failed to observe any group differences in overall response times (RTs), as well as in Stroop (incongruent vs. congruent) and in Incongruity (incongruent vs. neutral) effect sizes, for both Classic and Numerical Stroop tasks. These findings contribute to the larger picture that a bilingual advantage is not systematically found in control tasks and they suggest that we may still have much to learn about the diversity of bilinguals we are testing. However, these findings also indicate that if a bilingual advantage is found in a Stroop task, it is not straightforward to explain it with reduced L1 language activation in bilinguals [cf. the *weaker links hypothesis* by Gollan et al. (2005)]. More importantly, the overall RT advantage in bilinguals compared to monolinguals on both congruent and incongruent trials seriously questions the conclusion that multiple language use may specifically improve performance in tasks presenting a conflict (see Hilchey and Klein, 2011 for a review). This overall RT advantage in some bilingual individuals suggests that these bilinguals are not better in conflict resolution in particular but rather that they may have either a “bilingual executive processing advantage” as proposed by Hilchey and Klein (2011) or a general enhanced capacity of processing information independently of the presence of conflicting information. The more general question we are asking here is whether there is a relationship between the use of multiple languages and the improvement of executive control efficiency, at least at some stages of second-language learning, or, more specifically, which kinds of control processes are improved by multiple language use. This assumption relates to Hilchey and Klein (2011) who claimed that many executive processes show a bilingual benefit, though not necessarily inhibition. In this paper, we will provide evidence for very specific bilingual benefits with respect to sub-processes of cognitive control.

To account for inconsistencies observed in the literature of bilingualism and executive functions, various methodological considerations can also be invoked. One of them is that until now most of the studies have used RTs as the dependent variable, which are known to result from a combination of multiple processes and sub-processes. In the present study, we recorded online electrical responses of the brain in order to trace the precise time course of the two sub-processes of interference control under investigation, namely conflict monitoring and interference suppression and their neural underpinnings. More particularly, we recorded event-related potentials (ERPs) and associated neuronal generators of ERP signatures while a group of French–German participants and their matched monolingual controls performed a Stroop task combined with a Negative priming paradigm. To study cognitive inhibition, and more particularly the overcoming of inhibition, the Negative priming paradigm, initially implemented in a Stroop task by Dalrymple-Alford and Budayr (1966), constitutes a suitable tool (Aron, 2007; for a review and for alternative explications of the Negative priming effect, see MacLeod and MacDonald, 2000). The inconsistencies observed in the literature of bilingualism and executive functions can also be the result of considering bilingualism as a categorical variable, thus masking the impact of the multiple dimensions characterizing bilingual individuals. In the present study, we used correlation analyses to embrace the multidimensional facets of bilingualism.

Over the past 20 years in cognitive psychology, neurophysiological and neuroimaging techniques have demonstrated their capacity to detect effects on a more fine-grained scale than various behavioral methods. In research on executive functions in monolinguals, three ERP signatures have been established repeatedly using different tasks. From a neurochronometric point of view, the first signature is the fronto-central N200 effect (i.e., a larger negative amplitude in the conflict compared to the non-conflict condition) assumed to reflect cognitive control (response inhibition, response conflict, and error monitoring; Boenke et al., 2009), and whose main neuronal generator was found in the ACC (Folstein and Van Petten, 2008). The second ERP signature is the centro-parietal N400 effect, usually found in Stroop studies (i.e., a larger negativity in the incongruent condition in comparison to the congruent or to the neutral condition; Liotti et al., 2000; West, 2003; Hanslmayr et al., 2008; Appelbaum et al., 2009; Bruchmann et al., 2010; Coderre et al., 2011; Naylor et al., 2012; among others). The N400 Stroop interference was interpreted to reflect higher cognitive cost in responding to stimuli in the incongruent condition – usually causing a conflict between the two sources of information, the color word and the print color – in comparison to the congruent condition. The main neuronal generators of the N400 effect were mainly found in both, the ACC and the prefrontal cortex (PFC; Liotti et al., 2000; Markela-Lerenc et al., 2003; Hanslmayr et al., 2008; Bruchmann et al., 2010). Finally, a later ERP signature was also observed, namely a late sustained negative-going potential (540–700 ms), that is a sustained fronto-central negative deflection in the incongruent condition compared to the congruent one (West, 2003; Hanslmayr et al., 2008; Naylor et al., 2012). Note that some studies also reported the inverse effect: a positive deflection, over the centro-parietal scalp (Liotti et al., 2000; West, 2003; Hanslmayr et al., 2008; Appelbaum et al., 2009; Coderre et al., 2011). The late sustained negative-going potential has been proposed to reflect either engagement of executive processes (Hanslmayr et al., 2008), conflict resolution processes (Coderre et al., 2011; Naylor et al., 2012), semantic reactivation of the meaning of words following conflict resolution (Liotti et al., 2000; Appelbaum et al., 2009), or response selection (West, 2003). Source localization has rarely been done for this late sustained negative-going potential but there is some evidence of its main neuronal generators in the middle or inferior frontal gyrus and the extrastriate cortex (West, 2003).

Recently, in an ERP study examining the impact of bilingualism on interference suppression, using Stroop, Simon, and Erikson flanker tasks, Kousaie and Phillips (2012) reported language group differences in conflict processing at the neurophysiological level (i.e., larger fronto-central N200 amplitudes and later P3 peak latencies for mono- than for bilinguals in a Stroop task) but not at the behavioral level. This finding suggests that neurophysiological measures can be more sensitive than behavioral measures. Moreover, in an ERP study also using a Stroop task, Coderre and van Heuven (2014) found a descriptively smaller N400 effect in bilinguals compared to monolinguals. In an MEG study using a Simon task, Bialystok et al. (2005) reported different correlations between the brain areas activated and the reaction times comparing bi- and monolinguals, indicating systematic differences in the activation of cognitive control areas (e.g., PFC, ACC) between the two language groups. In general, the positive correlation of faster reaction times with stronger activation in PFC and ACC in bilinguals corroborates the idea that bilingualism is associated with plasticity in cognitive control efficiency. Regarding the neuronal sources underlying bilingual language control, Abutalebi and Green (2008; see also, Green and Abutalebi, 2013) formulated a neurocognitive model constituted by a cerebral network including the ACC, the PFC, the basal ganglia (especially the caudate nucleus; see also, Crinion et al., 2006), the bilateral supramarginal gyri (SMG) and the parietal lobe (in case of high attentional load). Note that this model is widely coherent with neurocognitive models of domain-general control (MacDonald et al., 2000; Shenhav et al., 2013).

The present ERP study relies on an integrative theoretical account, i.e., the *Adaptive Control Hypothesis* model postulating that various control processes are involved in use of multiple languages (Green and Abutalebi, 2013). Our goal was to investigate the impact of bilingual experience on the neurodynamics of distinct control processes, i.e., conflict monitoring, interference suppression, overcoming of inhibition, and conflict resolution by combining a Stroop task with a Negative priming paradigm and using a high temporal resolution technique, namely electroencephalography (EEG). The experiment was administered to 22 late non-balanced French–German bilinguals and 22 French monolinguals. A correlation statistical approach, in which multiple dimensions inherent to bilingualism (i.e., linguistic, environmental, and demographic dimensions) were treated as continuous variables, was adopted to take into consideration the non-categorical nature of bilingualism.

Based on previous studies, an N200 effect (conflict detection/conflict monitoring), an N400 effect (interference suppression) and a late sustained negative-going potential (conflict resolution) should be observed for both the Stroop and Negative priming tasks. For bilinguals, smaller effect sizes were expected in the three time windows for the Stroop task and even more so for the more costly Negative priming task, when compared to monolinguals. Finally, and critically, based on current assumptions on the functional relationship between ACC and PFC (MacDonald et al., 2000), we hypothesized that ACC should monitor conflict and then communicate with PFC for implementation of control once the need has been identified. Thus, we predicted to find ACC activation especially for the early N200 and the N400 effect while PFC activation was supposed to mainly underlie the N400 component and the late sustained negative-going potential.

## Materials and Methods

### Participants

Forty-four right-handed (Edinburgh Handedness Inventory) participants were selected for the experiment and tested at Paris Descartes University, France. Among them were 22 successive French (L1) – German (L2) bilinguals and 22 French monolingual individuals, all of them living in France at the time of the experiment. The study was approved by the *Conseil d’évaluation éthique pour les recherches en santé* at Paris Descartes University and participants gave their written informed consent prior to participation. By their own account, participants had no history of current or past neurological or psychiatric illnesses, had normal or corrected-to-normal vision and normal color vision. They were paid 10€ per hour or received course credits for their participation. As it has been pointed out in previous studies demographic factors such as socioeconomic status (SES; Morton and Harper, 2007) and environmental factors such as expertise in music (Bialystok and DePape, 2009), video game playing (Dye et al., 2009), and actively performing sports requiring high bimanual coordination (Diamond and Lee, 2011) are all critical factors for developing executive control mechanisms. Consequently, these factors were controlled in our study (see **Table 1**).

**Table 1:** Language background and environmental factors.

	Bilinguals (*n* = 22)		Monolinguals (*n* = 22)		*p*
	Mean	SD	Mean	SD	
Age [years]	26.9	(5.5)	25.5	(4.4)	n.s.
Freq. of daily language use other than L1 [%]	26.6	(13.9)	0.6	(0.9)	<0.001
Freq. of daily L2 use [%]	21.4	(14.1)	0.5	(0.8)	<0.001
Freq. of daily L3 use [%]	4.8	(6.4)	0.2	(0.6)	<0.01
L2 PS [1: high – 5: low]	1.7	(0.6)	–	–	–
L2 PO [%]	83.0	(9.5)	–	–	–
Music practice [hour/week]	0.9	(2.2)	1.1	(2.2)	n.s.
Sport practice [hour/week]	1.6	(2.1)	1.4	(2.0)	n.s.
Vid/Comp game play [hour/week]	0.9	(2.1)	2.1	(4.4)	n.s.

*Results from the evaluation of the participants’ language background and environmental factors such as music practice, high coordination sports, or computer/video game play are shown. Mean and SD are indicated for each factor. Freq., frequency; L2 PO, proficiency in L2 German – objective (Test: DAF – *Deutsch als Fremdsprache*); L2 PS, proficiency in L2 German – subjective (self-evaluation on a scale from 1 – *high proficiency* to 5 *– low proficiency*); Vid/Comp game play, Video and Computer game play.*

Twenty-two successive French (L1)-German (L2) bilinguals (16 female) of an average age of 26.9 ± 5.5 years (range = 18–36 years) were tested. They were late learners of German who had started to study German from the age of 10 at secondary school in France. The mean AoA of their second language (L2) was 10.6 ± 0.7 years (range = 9–12 years). Bilingual participants had a regular use of their L2 German during the past 3 years and at present (20.9 ± 14.6% per day; see **Table 1**) and even if they were highly proficient in their L2 [self-evaluation; 1.7 ± 0.6 (1 – *high proficiency* to 5 *– low proficiency*); score language test: 83.0 ± 9.5%] they were non-balanced bilinguals. Language background data assessed with a language history questionnaire are summarized in **Table 1**.

Twenty-two monolingual French native speakers (13 female) of an average age of 25.5 ± 4.4 years (range = 19–39 years) who had had little use of languages other than their L1 during the past 3 years and at present (0.6 ± 0.9% per day; see **Table 1**) were selected as the monolingual control group.

### Stimuli

An adapted version of the original Stroop task (Stroop, 1935) was used in the experiment. The task consisted of manually responding to the print color of stimuli in four different conditions, namely congruent, incongruent, negative priming, and neutral. In the congruent condition, the meaning of the color word and the print color matched (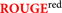), while in the incongruent and negative priming conditions they did not (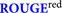). In the negative priming condition, an incongruent stimulus (trial *n*) was preceded by an incongruent trial (trial *n*-1) serving as the negative prime: in trial *n*-1 the color word that had to be inhibited (‘red’ in 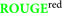) was equal to the print color which was to name in trial *n* (‘red’ in 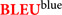). Therefore, the inhibition affecting the color ‘red’ in trial *n*-1 needed to be overcome to correctly respond to the print color in trial *n*. In the congruent, incongruent and negative priming conditions, the following four color words were presented in L1, French: ROUGE^red^, BLEU^blue^, JAUNE^yellow^, VERT^green^ and their translation equivalents in L2, German: ROT^red^, BLAU^blue^, GELB^yellow^, GRÜN^green^. In the neutral condition, four non-color words were presented in the same print colors as in the congruent and incongruent conditions (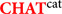) in L1, French: CHAT^cat^, CHIEN^dog^, MAIN^hand^, PIED^foot^ and their translation equivalents in L2, German: KATZE^cat^, HUND^dog^, HAND^hand^, FUSS^foot^. The stimulus words, written in capitals of font “Calibri” in font size 48, were presented individually against a black background in the center of the screen.

### Procedure

**FIGURE 1:**
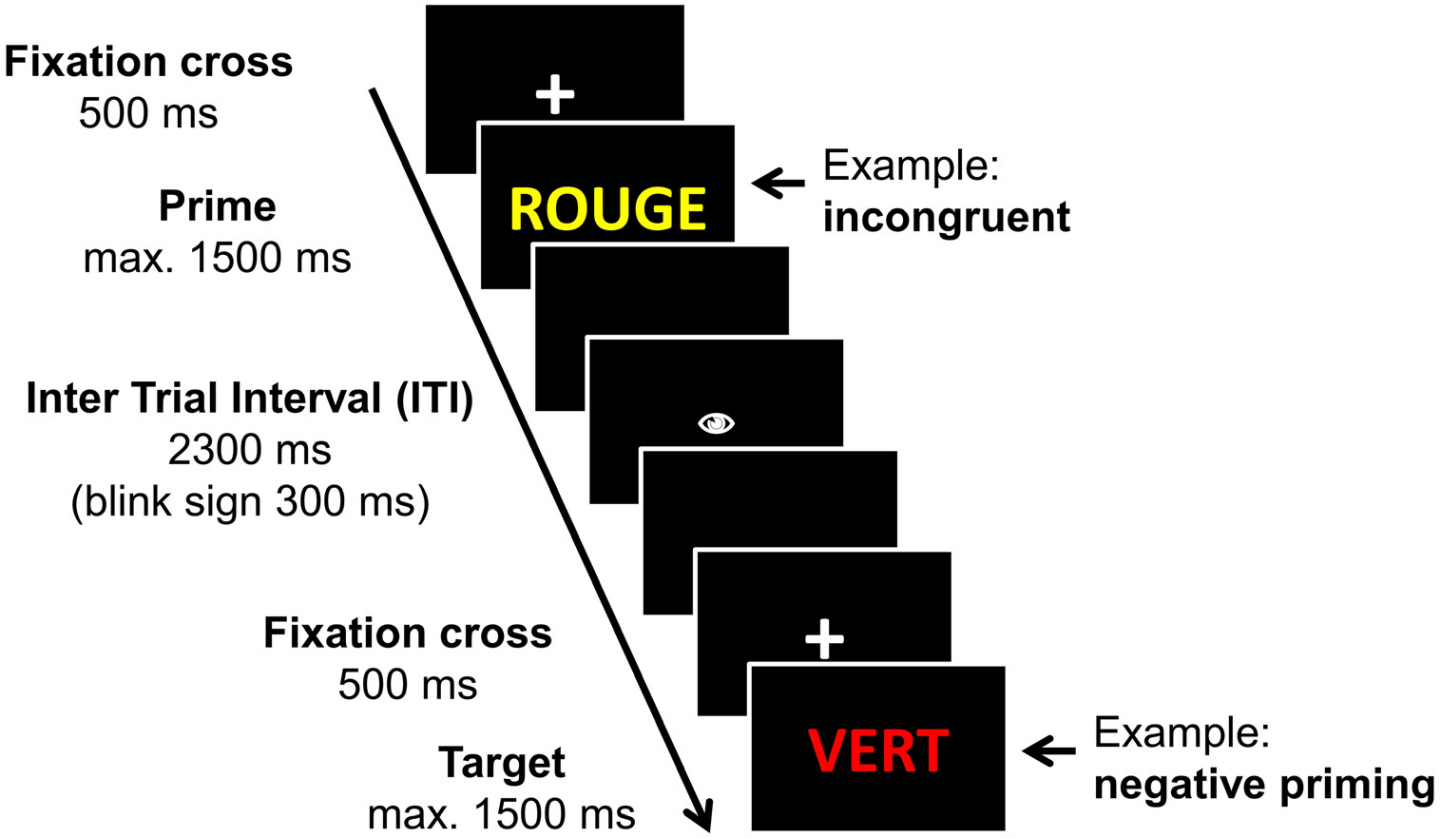
**Timing of a trial.** The timing of a trial in the manual version of a Stroop task is displayed. Two succeeding trials are given in order to demonstrate the Negative priming procedure.

Participants were seated in front of the computer screen (14” screen) and instructed to perform a manual color response task, that means they had to indicate as fast and as correctly as possible the print color of the stimulus word by pressing one of the four color-coded response buttons (keys *d*, *f*, *j*, and *k*). The color-finger-assignment was counterbalanced between-subjects. Stimuli were presented with *E-Prime 1.2* (Psychological Software Tools, Pittsburgh, PA, USA). Each trial started with a fixation-cross presented in the center of the screen for 500 ms (**Figure 1**), which was then replaced by the stimulus word. The stimulus remained visible until one of the four color response keys was pressed (online RT) but maximally for 1500 ms. Then followed an inter trial interval (ITI) of 2300 ms figuring a black screen. After the first 1000 ms of the ITI, a blink sign (a symbolized eye) was displayed for 300 ms. Participants were instructed to limit eye blinks to the interval starting with the blink sign until the end of the ITI in order to reduce motor artifacts on the ERP response.

In order to enable the participants to learn the color-key correspondences, two training blocks of 40 trials each were presented before starting the ten experimental blocks. If accuracy was below 80% after the second training block, training was repeated. For bilinguals, five experimental blocks featured words in German, the five other blocks featured words in French. For monolinguals all blocks consisted of words in French but only five were selected for further analysis. In order to compare Language groups, only the procedure for L1 (French) blocks is presented as follows. Each block consisted of 72 trials, consisting of 24 congruent, 12 incongruent, 12 negative priming and 24 neutral stimuli, presented in a pseudo-randomized order. Online RT was defined as the interval between the onset of the stimulus word and the button press. Responses before 200 ms or after 1500 ms were coded as missing. We averaged the RTs for correct responses for each experimental condition across participants and across items. RTs outside a range of 2 standard deviation from the mean per participant were excluded from the statistical analysis.

### Analysis of Behavioral Data

Two-way repeated measures analysis of variances (ANOVAs), including the within-subjects factor Condition (congruent, incongruent, negative priming, neutral) and the between-subjects factor Language group (bilingual, monolingual) were conducted for the dependent variables Error rate and RT. Moreover, in order to compare the behavior in the two languages of the bilingual participants, two-way repeated measures ANOVAs were conducted including the within-subjects factors Condition (congruent, incongruent, negative priming, neutral) and Language (L1, L2) for the dependent variables Error rate and RT.

### ERP Recording

Electroencephalography was recorded using a Geodesics 64-channel sensor net and the software NetStation (Electrical Geodesics Inc., Eugene, OR, USA). All channels were referenced online against Cz. For data analysis, channels were re-referenced to an average reference. Electrode impedances were kept below 50 kΩ. Data were recorded at a sampling rate of 500 Hz, with an online 0.1–80 Hz frequency bandpass filter. Then, data were filtered offline with a 0.5–35 Hz bandpass filter.

### ERP Analysis

The continuous EEG were segmented into epochs from 200 ms pre-stimulus until 1500 ms post-stimulus onset and baseline corrected with the baseline set from -200 to 0 ms. Only trials with correct responses that were not contaminated by ocular or other movement artifacts were kept for further data analysis. Automatic detection was run followed by a visual inspection of the segmented data. The total percentage of rejected trials were distributed equally over the four conditions (*F* < 1; congruent: 37.3 ± 16.9%, neutral: 38.3 ± 15.4%, incongruent: 38.0 ± 16.9%, negative priming: 37.6 ± 16.1%). This is true for rejected trials due to erroneous behavioral responses (congruent: 3.2 ± 3.0%, neutral: 3.3 ± 3.5%, incongruent: 3.8 ± 4.2%, negative priming: 3.6 ± 3.8%) as well as due to artifacts in the signal (congruent: 34.0 ± 16.7%, neutral: 35.0 ± 14.9%, incongruent: 34.1 ± 16.4%, negative priming: 34.0 ± 15.6%). In each experimental condition, the ERP activity was then averaged over stimuli and over participants (i.e., grand average ERP). Statistical analyses were conducted for three ERP signatures for which the time windows were selected based on previous ERP studies of executive functioning and adjusted by visual inspection of the grand averages: N200 (200–300 ms), Stroop N400 (400–500 ms), and a late sustained negative-going potential (540–700 ms). For the three selected intervals, analyses were conducted on the ERPs from selected electrodes. All analyses were quantified using the multivariate approach to repeated measurement and followed a hierarchical analysis schema. In order to allow for an examination of hemispheric differences, the data recorded at the lateral recording sites were treated separately from the data recorded at the midline electrode sites. Analyses are presented for the Stroop effect (incongruent vs. congruent condition) and the Negative priming effect (negative priming vs. congruent condition) because our hypotheses were centered on these effects.

For the lateral recording sites, for each time window, a four-way repeated measures ANOVA including the within-subjects factors Condition (Stroop: incongruent, congruent; Negative priming: negative priming, congruent), the topographical variables Hemisphere (left, right) and Region (anterior, posterior) and the between-subjects factor Language group (bilingual, monolingual) was conducted. Four regions of interest (ROIs) resulting from a complete crossing of the Region and Hemisphere variables were defined: left anterior (F7, F3, FT7, FC3), right anterior (F8, F4, FT8, FC4), left posterior (CP5, P7, P3, O1), and right posterior (CP6, P8, P4, O2).

For the midline electrodes, a three-way repeated measures ANOVA including the within-subjects factors Condition (Stroop: incongruent, congruent; Negative priming: negative priming, congruent), Electrode (Fz, Cz, Pz) and the between-subjects factor Language group (bilingual, monolingual) was run for each of the three time windows of interest. Moreover, given that we had a hypothesis on differences between Language groups based on previous studies, two-way repeated measures ANOVAs including the factors Condition (Stroop: incongruent, congruent; Negative priming: negative priming, congruent), and Language group (bilingual, monolingual) were run on each of the three midline electrodes in each time window. The dependent variable was the voltage amplitude averaged over each interval of interest. The Greenhouse–Geisser correction (Greenhouse and Geisser, 1959) was applied when evaluating effects with more than 1 degrees of freedom in the numerator. *Post hoc* pairwise comparisons at single electrode sites were performed using a modified Bonferroni procedure (Keppel, 1991). A significance level of 0.05 was used for all statistical tests and only significant results are reported.

### Source Analyses

Hanslmayr et al. (2008) proposed a dipole (localizing neuronal source activity) model for a Stroop task containing eight discrete dipoles in fixed locations: LOC/ROC (visual stimulus processing), LMC/RMC (manual response), ACC (cognitive control), LMTC (color processing), LPFC/RPFC (cognitive control). This eight dipoles model is based on theoretical assumptions of cognitive processes and their neural correlates involved in the execution of a Stroop task and has been tested and partially confirmed by Bruchmann et al. (2010). Here, we applied a 10-regional sources model including the sources proposed by Hanslmayr et al. (2008) plus two further neuronal generators found to be involved in Stroop processing, that is the LIFG/RIFG (cognitive control, inhibition; Peterson et al., 2002), in order to capture the largest number of neuronal sources (**Table 2**). Due to heterogeneous findings of peak activation in the ACC for a Stroop task in the previous literature and in order to improve the variance explained by the source model, the coordinates for the regional source in the ACC were chosen from a meta-analysis on a Stroop task (Laird et al., 2005).

**Table 2:** Source localization coordinates.

Brain region	Abbrevations	BA	Talairach coordinates			Reflected process
			*x*	*y*	*z*	
Left occipital cortex	LOC	17	−21	−79	-1*	Visual stimulus processing
Right occipital cortex	ROC	17	21	−79	−1*	Visual stimulus processing
Left medio-temporal cortex	LMTC		−45	−55	9*	Color processing
Left motor cortex	LMC	4	−36	−17	59*	Manual response
Right motor cortex	RMC	4	36	−17	59*	Manual response
Anterior cingulate cortex	ACC	32	1	16	38^Δ^	Cognitive control, attention, motor modulation, response selection
Left prefrontal cortex	LPFC	46/6	-32	22	57*	Cognitive control
Right prefrontal cortex	RPFC	46/6	32	22	57*	Cognitive control
Left inferior frontal gyrus	LIFG	44	−45	7	14†	Cognitive control, inhibition
Right inferior frontal gyrus	RIFG	44	44	8	13†	Cognitive control, inhibition

For discrete source localization of scalp ERPs in a Stroop task, Talairach coordinates have been taken from the following studies: *Hanslmayr et al., 2008; ^Δ^Laird et al., 2005; †Peterson et al., 2002; For extended regions, coordinates for peak activation specifically found in a Stroop task are indicated. BA, Brodman area.

In the present study, discrete source analysis was done with the Brain Electrical Source Analysis program (BESA, version 5.3., Megis Software, Heidelberg, Germany). Regional sources were seeded in fixed locations while their orientations were a free parameter. This theoretical model of regional sources explained 75.3% of the variance. In order to trace the neuronal generators of scalp ERP effects, statistical analyses using bootstrap confidence intervals (99%) were conducted using BESA (version 5.3.) and the Waveforms toolbox for Matlab. The bootstrapping procedure was applied to investigate source activation underlying the Stroop effect (incongruent vs. congruent) and the Negative priming effect (negative priming vs. congruent) on each neuronal source in our theoretical model (ACC, LPFC, RPFC, LIFG, RIFG, LMC, RMC, LOC, ROC, LMTC). The source ERP amplitude between two conditions was considered to be significantly different (*p* < 0.01) for intervals in which the confidence interval (99%) of the difference wave did not include zero.

### Correlation Analyses

As we consider that taking bilingualism as a categorical variable and therefore conducting ANOVAs is a necessary but not a sufficient approach to explore the impact of bilingualism on neuronal measures of cognitive control, we additionally conducted correlation analyses between linguistic background measures and behavioral and neurophysiological Stroop and Negative priming effect^1^ sizes in bilinguals, with the following factors: the frequency of L2 and of L3 use, L2 proficiency, duration of immersion in an L2 environment, and age of immersion.

## Results

### Behavioral Results

**FIGURE 2:**
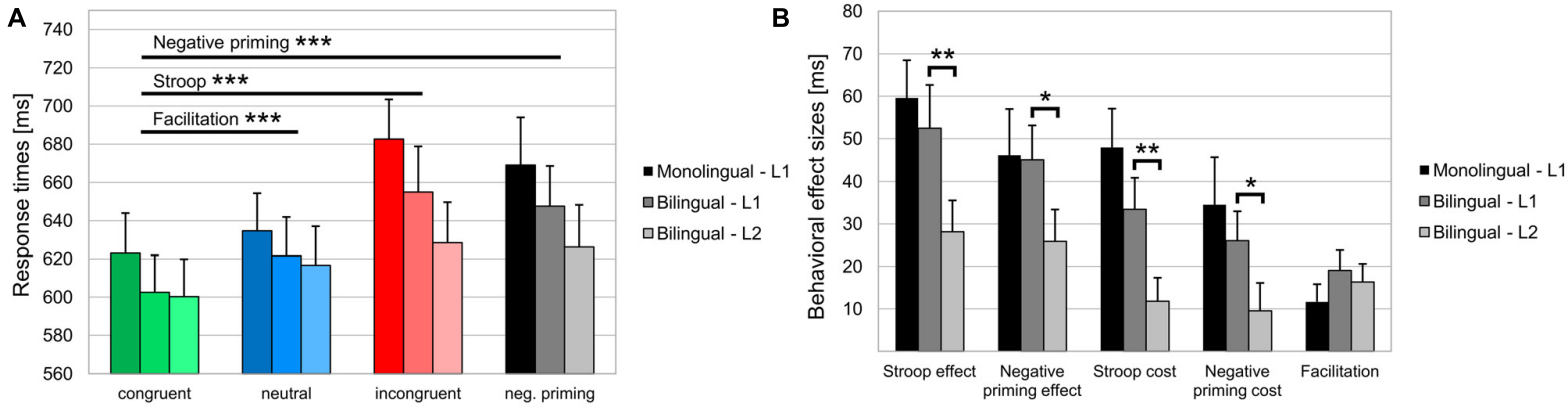
**Behavioral data. (A)** Mean response times [ms] and the SEM in the congruent, neutral, incongruent and negative priming experimental conditions and **(B)** behavioral effect sizes are displayed for the two Language groups in their L1 and for bilinguals also in their L2. ERR, error rate; RT, response time. *p < 0.05; **p < 0.01; ***p < 0.001.

Behavioral data are shown in **Table 3** and **Figure 2**. For Error rates, the two-way repeated measures ANOVA did not reveal a main effect of Condition (*F* < 1), nor a main effect of Language group (*p* > 0.10), or a Condition by Language group interaction (*F* < 1). For RTs, the ANOVA showed a main effect of Condition [*F*(3,126) = 38.54, MSE = 1099.94, *p* < 0.001, $\eta_{p}^{2}$ = 0.479], reflecting that RTs were longer in the incongruent (669 ± 105 ms) compared to the congruent condition [613 ± 94 ms; Stroop effect; *F*(1,42) = 68.6, MSE = 2013.8, *p* < 0.001, $\eta_{p}^{2}$ = 0.620] as well as compared to the neutral condition [628 ± 93 ms; *F*(1,42) = 61.97, MSE = 1660.6, *p* < 0.001, $\eta_{p}^{2}$ = 0.596]. Moreover, RTs were longer in the negative priming (658 ± 107 ms) compared to the congruent condition [613 ± 94 ms; Negative priming effect; *F*(1,42) = 45.8, MSE = 1999.8, *p* < 0.001, $\eta_{p}^{2}$ = 0.522] as well as compared to the neutral condition [628 ± 93 ms; *F*(1,42) = 21.12, MSE = 1908.5, *p* < 0.001, $\eta_{p}^{2}$ = 0.335]. RTs were shorter in the congruent (613 ± 94 ms) compared to the neutral condition [628 ± 93 ms; Facilitation effect; *F*(1,42) = 23.61, MSE = 439.8, *p* < 0.001, $\eta_{p}^{2}$ = 0.360]. Finally, there was no main effect of Language group (*F* < 1) nor a Condition by Language group interaction (*F* < 1).

**Table 3:** Behavioral data.

	Monolinguals		Bilinguals				Monolinguals		Bilinguals			
	L1 – ERR [%]		L1 – ERR [%]		L2 – ERR [%]		L1 – RT [ms]		L1 – RT [ms]		L2 – RT [ms]	
	Mean	SD	Mean	SD	Mean	SD	Mean	SD	Mean	SD	Mean	SD
Congruent	2.4	(2.1)	3.5	(3.4)	3.8	(3.2)	623	(98)	603	(91)	600	(92)
Incongr.	3.0	(2.3)	3.8	(4.3)	4.7	(5.3)	683	(98)	655	(112)	629	(100)
Neg. prim.	3.0	(2.3)	4.2	(4.5)	3.6	(4.3)	669	(116)	648	(99)	627	(104)
Neutral	2.4	(2.3)	3.9	(4.4)	3.6	(3.8)	635	(92)	622	(95)	617	(96)

*Error rates (ERR) [%] and response times (RTs) [ms] in the four experimental conditions (congruent, incongruent, negative priming, neutral) are indicated with standard deviation (SD) in parentheses for the two Language groups in their L1 and for bilinguals also in their L2. ERR, error rate; RT, response time; SD, standard deviation.*

Comparing the behavioral data in the L1 and L2 of bilinguals no differences were found for error rates. For RTs, however, there was a main effect of Language [*F*(1,21) = 4.44, MSE = 1879.4, *p* < 0.05, $\eta_{p}^{2}$ = 0.175] indicating that averaged RTs were shorter in L2 (618 ± 96 ms) compared to L1 (632 ± 97 ms). There was also a Condition by Language interaction [*F*(3,63) = 6.64, MSE = 337.04, *p* < 0.01, $\eta_{p}^{2}$ = 0.240] indicating that the Stroop effect (incongruent vs. congruent) was larger in L1 (52 ± 48 ms) compared to L2 (28 ± 35 ms; *p* < 0.01); furthermore, the Negative priming effect (negative priming vs. congruent) was larger in L1 (45 ± 38 ms) compared to L2 (26 ± 35 ms; *p* < 0.05). Finally, the *post hoc* analyses also showed that the Stroop cost (incongruent vs. neutral) was larger in L1 (33 ± 35 ms) compared to L2 (12 ± 26 ms; *p* < 0.01); similarly, the Negative priming cost (negative priming vs. neutral) was larger in L1 (26 ± 32 ms) compared to L2 (10 ± 31 ms; *p* < 0.05).

### Electrophysiological Results

#### Stroop Effect

In the time-window 200–300 ms, neither the four-way ANOVA on lateral electrodes nor the three-way ANOVA on midline electrodes revealed any main effect or interaction involving the factors Condition or Language group. Two-way repeated measures ANOVAs on each of the midline electrodes did not reveal any Condition by Language group interaction or main effect of Language group.

**FIGURE 3:**
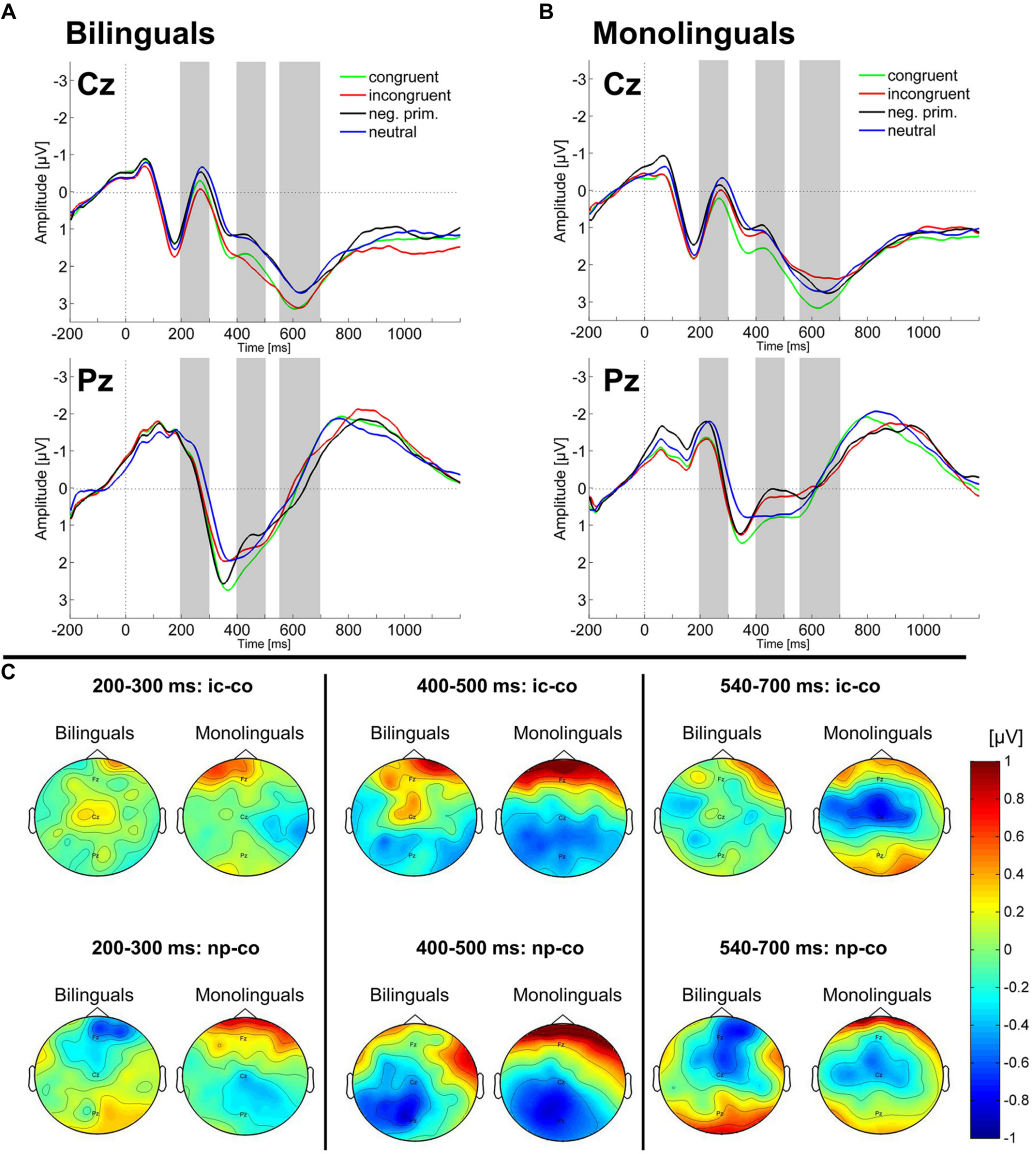
**Scalp ERPs.** The ERP in the four experimental conditions (congruent, neutral, incongruent, and negative priming) on the Cz and Pz electrodes are plotted for **(A)** bilinguals and **(B)** monolinguals. The gray bars indicate the time intervals which were used for statistical analysis of the mean amplitude. **(C)** Topographies of amplitude differences [μV] for the Stroop effect (incongruent – congruent; ic-co) and the Negative priming effect (negative priming – congruent; np-co) are shown for bilinguals and monolinguals in each of the three time windows.

In the time window 400–500 ms, the four-way ANOVA on lateral electrodes revealed a main effect of Condition [*F*(1,42) = 13.46, MSE = 0.15, *p* < 0.001, $\eta_{p}^{2}$ = 0.243] reflecting a more negative amplitude in the incongruent compared to the congruent condition (Stroop effect). Moreover, a Condition by Region interaction [*F*(1,42) = 5.98, MSE = 0.85, *p* < 0.05, $\eta_{p}^{2}$ = 0.125] indicated that the N400 Stroop effect was observed over the posterior scalp. Similarly, the three-way ANOVA on midline electrodes revealed a Condition by Electrode interaction [*F*(2,84) = 4.11, MSE = 1.5, *p* < 0.05, $\eta_{p}^{2}$ = 0.089] indicating that the N400 Stroop effect (incongruent more negative than congruent) was only significant at the Pz electrode [*F*(1,43) = 6.43, MSE = 0.75, *p* < 0.05, $\eta_{p}^{2}$ = 0.130]. Two-way repeated measures ANOVAs revealed a Condition by Language group interaction on the Cz electrode [*F*(1,42) = 4.57, MSE = 0.59, *p* < 0.05, $\eta_{p}^{2}$ = 0.098], reflecting that the N400 Stroop effect was significant for monolinguals (*p* < 0.05; **Figure 3B**) but not for bilinguals (*p* > 0.10; **Figure 3A**; see also **Figure 3C**). Given that we had a strong hypothesis on the modulation of the N400 effect between the two groups based on previous studies, we then conducted further two-way ANOVAs on electrodes neighboring the Cz electrode to determine whether the N400 effect was significant over other electrodes. These analyses revealed a significant Condition (incongruent, congruent) by Language group (bilingual, monolingual) interaction also on the electrode C1. A small ROI including the electrodes Cz and C1 was created and we conducted a three-way ANOVA [Condition (incongruent, congruent), Electrode (Cz, C1), Language group (bilingual, monolingual)] which revealed a significant Condition by Language group interaction [*F*(1,42) = 5.92, MSE = 0.804, *p* < 0.05, $\eta_{p}^{2}$ = 0.123]. *Post hoc* analyses revealed that there was a tendency toward a significant effect of Condition over this ROI for monolinguals (*p* = 0.078) while it was not significant in bilinguals (*p* > 0.10).

In the time-window 540–700 ms, neither the four-way ANOVA on lateral electrodes nor the three-way ANOVA on midline electrodes revealed any main effect or interaction involving the factors Condition or Language group. Two-way repeated measures ANOVAs revealed a main effect of Condition on the Cz electrode, reflecting a more negative amplitude in the incongruent compared to the congruent condition [Stroop effect; *F*(1,42) = 5.69, MSE = 0.570, *p* < 0.05, $\eta_{p}^{2}$ = 0.119]. Moreover, a Condition by Language group interaction on the Cz electrode [*F*(1,42) = 4.7, MSE = 0.57, *p* < 0.05, $\eta_{p}^{2}$ = 0.101], indicated that the late sustained negative-going potential was only significant in monolinguals (*p* < 0.01; **Figures 3A–C**). To test whether the interaction effect between Condition and Language Group was significant over electrodes neighboring Cz, additional two-way ANOVAs were run. These analyses revealed a Condition by Language group interaction also on electrodes C1 and FC1. Creating a small ROI with these three electrodes we conducted a three-way ANOVA [Condition (incongruent, congruent), Electrode (Cz, C1, FC1), Language group (bilingual, monolingual)], which revealed a significant Condition by Language group interaction [*F*(1,42) = 6.77, MSE = 1.094, *p* < 0.05, $\eta_{p}^{2}$ = 0.139]; *Post hoc* analyses showed that the incongruent condition was significantly more negative compared to the congruent condition in monolinguals (*p* < 0.001) while there was no significant difference in bilinguals (*F* < 1).

**FIGURE 4:**
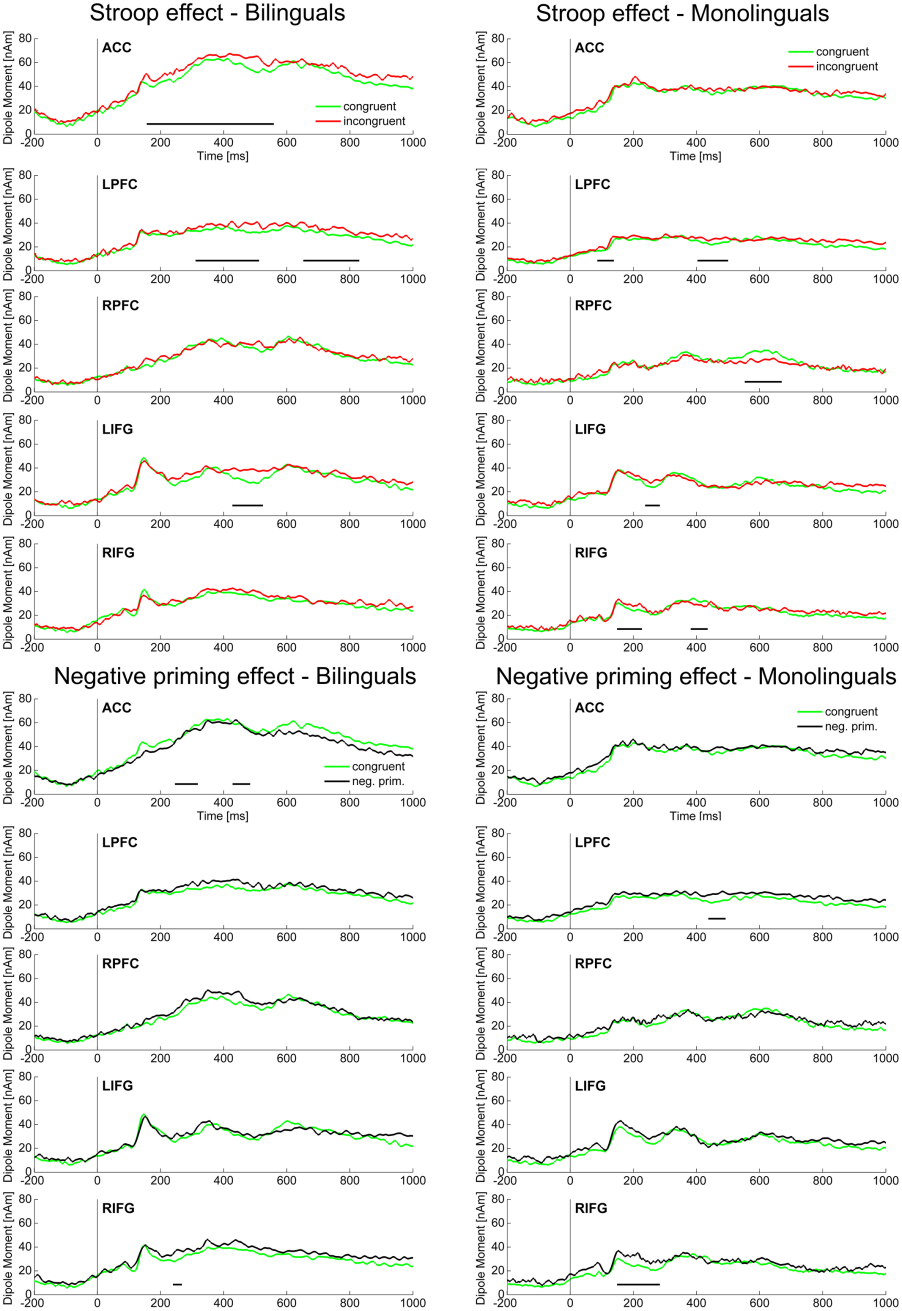
**Source ERPs**. Source activity in the ACC, LPFC, RPFC, LIFG, and RIFG is displayed for the Stroop effect (incongruent vs. congruent) and the Negative priming effect (negative priming vs. congruent) for the two Language groups in their L1. Intervals with a significant difference (*p* < 0.01) in source ERP amplitude between the two conditions are marked with a black bar. ACC, anterior cingulate cortex; LPFC, left prefrontal cortex; RPFC, right prefrontal cortex; LIFG, left inferior frontal gyrus; RIFG, right inferior frontal gyrus.

Source localization analyses collapsed over Language group (*n* = 44) revealed a significant difference between the incongruent and the congruent condition (Stroop effect) in the ACC (80–530 ms). In the PFC, however, the Stroop effect was significant later and only in the left hemisphere, LPFC (80–130, 300–530, 630–920 ms). Moreover, the Stroop effect was present in the following sources: LIFG (230–290 ms), RIFG (220–260 ms), RMC (230–270, 410–550 ms), LMTC (20–110, 160–240, 410–450 ms). Looking at the source activity of sources involved in control processes (ACC, LPFC, RPFC, LIFG, RIFG; **Figure 4**) groupwise, the following differences between bilinguals and monolinguals were found: in bilinguals the Stroop effect was significant (*p* < 0.01) in the ACC (160–570 ms), LPFC (310–520, 650–830 ms), and LIFG (420–530 ms). In monolinguals however, the Stroop effect was significant in LPFC (90–150, 400–510 ms), RPFC (560–680 ms), LIFG (250–300 ms), and RIFG (140–230, 380–440 ms) but not in the ACC.

#### Negative Priming Effect

In the time-window 200–300 ms, the four-way ANOVA on lateral electrodes did not show any main effect or interaction involving the factors Condition or Language group. The three-way ANOVA on midline electrodes revealed a significant Condition by Electrode by Language group interaction [*F*(2,84) = 3.9, MSE = 0.93, *p* < 0.05, $\eta_{p}^{2}$ = 0.085]. *Post hoc* analyses revealed a marginally significant Condition by Language group interaction on the Fz electrode [*F*(1,42) = 3.65, MSE = 0.79, *p* = 0.063, $\eta_{p}^{2}$ = 0.08] that was due to an effect inversion between Language groups (the negative priming condition being more negative compared to the congruent condition in bilinguals, while this effect was reversed in monolinguals). The two-way repeated measures ANOVAs on each of the three midline electrodes only revealed a main effect of Condition on the Cz electrode [*F*(1,42) = 8.17, MSE = 0.231, *p* < 0.01, $\eta_{p}^{2}$ = 0.163], reflecting a larger negativity in the negative priming condition compared to the congruent one (Negative priming effect).

In the time-window 400–500 ms, the four-way ANOVA revealed a significant Condition by Region interaction [*F*(1,42) = 14.63, MSE = 0.64, *p* < 0.001, $\eta_{p}^{2}$ = 0.258], indicating that the negativity was larger in the negative priming condition compared to the congruent one over the posterior electrodes. The three-way ANOVA on the midline electrodes revealed a main effect of Condition [*F*(1,42) = 9.84, MSE = 0.84, *p* < 0.01, $\eta_{p}^{2}$ = 0.190], in that the amplitude of the negativity in the negative priming condition was larger than the one found in the congruent condition. Moreover, there was a Condition by Electrode interaction [*F*(2,84) = 5.13, MSE = 1.59, *p* < 0.05, $\eta_{p}^{2}$ = 0.109] reflecting that the amplitude was more negative in the negative priming compared to the congruent condition on the centro-parietal electrodes Cz [*F*(1,43) = 12.8, MSE = 0.43, *p* < 0.001, $\eta_{p}^{2}$ = 0.230; **Figures 3A–C**] and Pz [*F*(1,43) = 16.4, MSE = 0.75, *p* < 0.001, $\eta_{p}^{2}$ = 0.276]. Two-way repeated measures ANOVAs did not reveal any main effect or interaction involving the factor Language group.

In the time-window 540–700 ms, the four-way ANOVA (Condition, Hemisphere, Region, Language group) revealed a significant Condition by Region interaction [*F*(1,42) = 5.32, MSE = 0.55, *p* < 0.05, $\eta_{p}^{2}$ = 0.112], indicating that over the anterior scalp, the negative priming condition was more negative compared to the congruent condition while over the posterior scalp the negative priming condition was more positive as compared to the congruent condition. The three-way ANOVA (Condition, Electrodes, Language group) revealed a significant main effect of Condition [*F*(1,42) = 5.55, MSE = 0.74, *p* < 0.05, $\eta_{p}^{2}$ = 0.117], indicating that the amplitude in the negative priming condition was more negative as compared to the congruent condition (**Figures 3A–C**). Two-way repeated measures ANOVAs on each of the three midline electrodes did not reveal any main effect or interaction involving the factor Language group.

Source localization analyses collapsed over Language group (*n* = 44) revealed a significant difference between the negative priming and the congruent condition (Negative priming effect) in the ACC (250–310, 440–490 ms). In the PFC, however, the Negative priming effect underlying the scalp ERP effects became significant later and only in the left hemisphere, LPFC (40–100, 420–490 ms). Moreover, the Negative priming effect was present in the following sources: RIFG (170–290 ms), LOC (250–360, 400–440 ms), RMC (450–550 ms). Looking at the source activity of sources involved in control processes (ACC, LPFC, RPFC, LIFG, RIFG; **Figure 4**) groupwise, the following differences between bilinguals and monolinguals were found: in bilinguals the Negative priming effect was significant (*p* < 0.01) in the ACC (240–320, 420–480 ms) and RIFG (250–280 ms). In monolinguals however, the Negative priming effect was significant in LPFC (430–490 ms) and RIFG (150–290 ms) but not in the ACC.

#### Correlation Analyses

**FIGURE 5:**
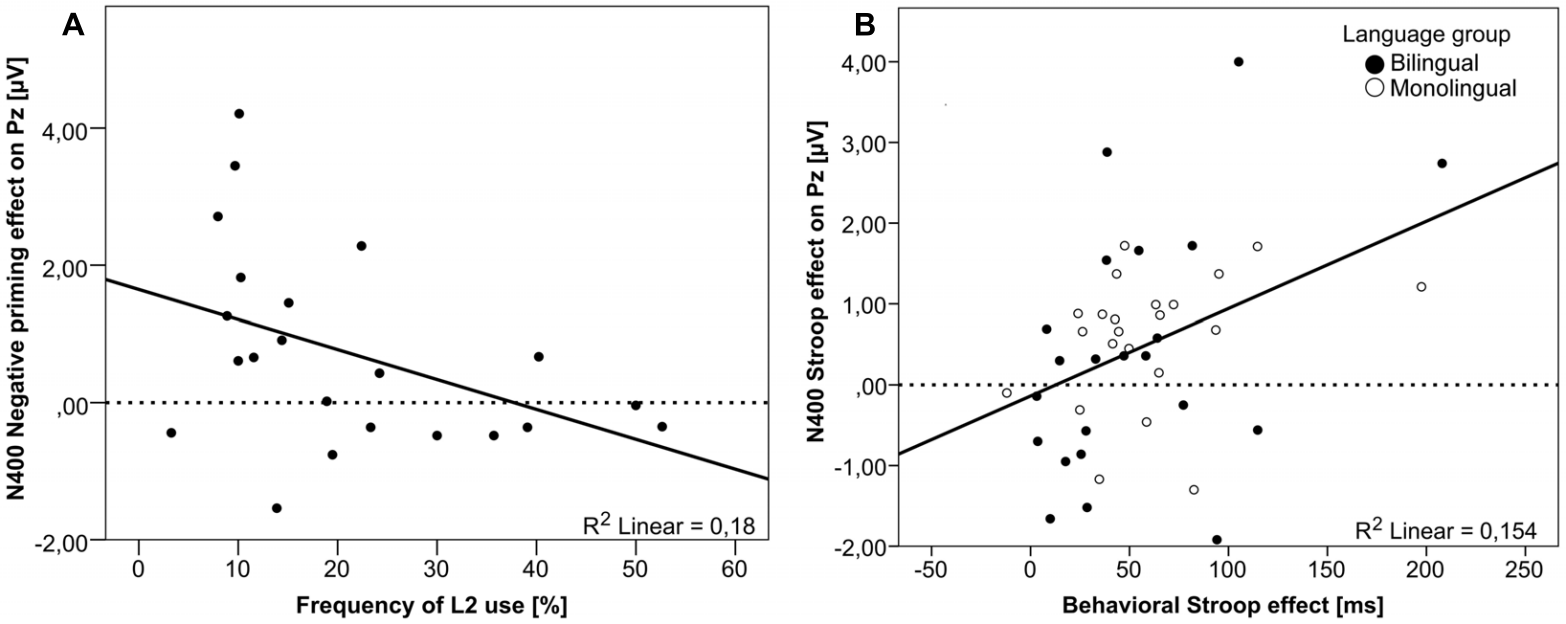
**Correlation analyses. (A)** The negative correlation between the Frequency of L2 use and the N400 Negative priming effect (congruent – negative priming) size in bilinguals (*n* = 22) on the Pz electrode, and **(B)** the positive correlation between the behavioral Stroop effect (incongruent – congruent) size and the neurophysiological N400 Stroop effect (congruent – incongruent) size on the Pz electrode (both Language groups collapsed; *n* = 44) are plotted.

One linguistic factor turned out to modulate the neurophysiological effect size in bilinguals: the frequency of L2 use was negatively correlated with the N400 Negative priming effect over the Pz electrode [*r*(22) = 0.424, *p* < 0.05; **Figure 5A**]. That means, the more bilinguals used their second language on a daily basis, the smaller was the N400 Negative priming effect.

## Discussion

The present study aimed to investigate the impact of bilingual experience on the neurochronometry of different control processes, i.e., control monitoring, interference suppression, overcoming of inhibition, and conflict resolution. For this purpose, a combined Stroop/Negative priming task was administrated to 22 late highly proficient but non-balanced French–German bilinguals and 22 French monolinguals while event-related brain potentials were recorded. At the neurophysiological level, a bilingualism benefit was found as revealed by reduced ERP effects in bilinguals in comparison to monolinguals, but this benefit was only observed in the Stroop task and was limited to the N400 and the late sustained potential ERP components. Moreover, and critically, we were able to show a differential time course of the activation of ACC and PFC in executive control processes. While the ACC showed major activation in the early time windows (N200 and N400) but not in the latest time window (late sustained negative-going potential), the PFC became unilaterally active in the left hemisphere in the N400 and late sustained negative-going potential time windows.

### Event-Related Potentials

On the neurophysiological level, three effects were expected: a central N200 effect (more negative amplitude in the negative priming and the incongruent conditions compared to the congruent condition in the 200–300 ms time window), a centro-parietal N400 effect (more negative amplitude in the negative priming and the incongruent conditions compared to the congruent condition in the 400–500 ms time window) and a fronto-centrally distributed late sustained negative-going potential (more negative amplitude in the negative priming and the incongruent conditions compared to the congruent condition in the 540–700 ms time window). We predicted to find reduced Stroop and Negative priming interference effects – reflecting reduced cost in conflict processing – in bilinguals compared to monolinguals.

An N200 effect was only observed for the Negative priming task (negative priming minus congruent). The increased negativity reported in the incongruent condition could be explained by an inhibition account (Aron, 2007) postulating that responses in a negative priming condition are usually delayed due to the necessity to overcome previously applied inhibition in order to access response-relevant information. However, note that we did not find longer latency in the incongruent condition in comparison with the congruent one. Hence, the N200 Negative priming effect may reflect overcoming of inhibition and/or high demand in conflict monitoring, which are processes that plausibly take place in negative priming trials but not in incongruent trials. Furthermore, an N400 effect was found for the Negative priming task (negative priming more negative than congruent; N400 Negative priming effect) as well as in the Stroop task (incongruent more negative than congruent; N400 Stroop effect). This observation replicates previous observations of a sensitivity of the N400 time window to Stroop interference (Liotti et al., 2000; Markela-Lerenc et al., 2003; Hanslmayr et al., 2008). Similarly, in the present study, the more negative N400 amplitude in the incongruent Stroop condition may reflect underlying inhibitory processes. Furthermore, consistent with previous findings the N400 effect was larger for the more costly task, i.e., Negative priming (N400 Negative priming effect; negative priming more negative than congruent).

The critical question of the present study concerned group differences: we observed smaller effect sizes for bilinguals in comparison with monolinguals but only for the N400 and the late sustained negative-going potential ERP effect in the Stroop task. No group difference was found in the early time window of the N200. It is plausible that a smaller Stroop N400 effect reflects reduced orthographic interferences that might be due to more efficient inhibition of interfering information. Similarly, Coderre and van Heuven (2014) have also reported a smaller Stroop N400 effect in bilinguals compared to monolinguals. Note that some authors label this incongruency effect P3 effect; for example Kousaie and Phillips (2012) found that the Stroop P3 peaked earlier in bilinguals as compared to monolinguals. Finally, a larger Stroop N400-like effect has been reported for children with learning disabilities as compared to age-matched controls, which was interpreted to reflect interference control deficits (Liu et al., 2014).

Correlation analyses between behavioral and neurophysiological effect sizes corroborate the idea that a smaller Stroop effect reflects better inhibitory capacities, in that an increasing behavioral Stroop effect was found to be reflected by an increasing N400 effect [at Pz electrode; *r*(44) = 0.393, *p* < 0.01; **Figure 5B**] in the present study. Concerning the reduced late sustained negative-going potential effect observed in the Stroop task for bilinguals as compared to monolinguals, it is not easy to find a good interpretation as there is a lack of consensus on the functional significance of this effect. Some authors have proposed that the late sustained negative-going potential may reflect stages of conflict resolution. Thus, the group differences we reported for the N400 and the late sustained negative-going potential might suggest that the bilinguals tested in our study may have less cost in dealing with the conflict present in a Stroop task. Taken together, for the Stroop task, a bilingual advantage has been found in the stages of conflict processing that are thought to reflect control implementation involving interference suppression (N400 effect) and conflict resolution (late sustained negative-going potential).

However, surprisingly, and against our predictions on task complexity, we failed to show both at the behavioral and neurophysiological levels a bilingual advantage in the Negative priming task, though considered a more complex task. Hence, the similarity of behavioral and electrical responses in the two groups in the Negative priming task could be an indicator that control processes specifically involved in this task may not be more efficient due to bilingual experience. Nonetheless, correlation analyses revealed a modulation of Negative priming effect size with frequency of L2 use (positive correlation), which indicates that bilingualism experience does have a certain impact on processes taking place in a Negative priming task, such as overcoming of inhibition, but that considering bilingualism as a categorical variable might not be sufficiently sensitive to capture this effect. Moreover, the heterogeneity in the monolingual group should not be neglected, in that ‘monolingual’ individuals nonetheless do have some basic foreign language experience – even if the extent was controlled to be as little as possible. This heterogeneity should, however, influence Stroop effects and Negative priming effects equally.

The differences between language groups observed for Stroop but not for Negative priming effect sizes, though unexpected, may actually corroborate the idea that the bilingual advantage in the Stroop task is mainly due to differences in control efficiency but not to the lower activation of the linguistic component in bilinguals. The *weaker links hypothesis* by Gollan et al. (2005) predicts similar effects for Stroop and Negative priming effects sizes. Thus, if the use of more than one language and consequently the reduced frequency of use of each single language in bilinguals were the main cause for their Stroop benefits, a comparable reduction of the effect size should have been observed for the Negative priming effect sizes in the present study, which was not the case. Consequently, the differences between the two language groups appear to be attributable to differences in the efficiency of specific control processes involved in the different tasks.

To account for the absence of a group difference for the neurophysiological N400 effect in the Negative priming task despite the observation of (1) a bilingualism advantage in the N400 Stroop task, i.e., a less complex task, (2) a negative correlation between frequency of L2 use and magnitude of the Negative priming N400 effect in bilinguals, and (3) a stronger involvement of ACC in bilinguals than in monolinguals, we propose the following interpretation: we suggest that the specificity of the experimental constraints imposed by the Negative priming design is playing a major role here. Whereas in the Stroop task, incongruent trials were equally preceded by congruent or by neutral trials, in the Negative priming paradigm, a negative priming trial was always preceded by an incongruent trial due to the rational of the paradigm (overcoming of an information that was inhibited in an incongruent previous trial). Thus, we propose that the absence of a group effect in the negative priming condition at the neurophysiological level could be due to the fact that the monolingual individuals were already in a mode of inhibition when they encountered a negative priming trial. Consequently, they benefited from a local advantage so that they were able to manage the complexity of the Negative priming task as well as the bilinguals. The bilinguals, on the other hand, may have benefited less from this local advantage as their inhibitory capacities are already at ceiling. This *post hoc* explanation of a finding that turned out to be inconsistent with our primary hypothesis of task complexity, may shed a new light on the functioning of control processes. Indeed, it suggests that when we put monolinguals in an inhibition mode, they become able to manage a complex control task as efficiently as bilinguals. This means that, at least at short-term, the executive control processes involved for performing the Negative priming task were sufficiently efficient in monolinguals for reaching the same level of control as that observed in bilinguals usually assumed to present an advantage in cognitive control. At least at short-term, an advantage may also be found in monolinguals when they work in an inhibition mode. This would argue for neurophysiological plasticity of the cognitive control processes under investigation in the present study. Further work should attempt to disentangle the respective role of second language use and mode of information processing on the improvement of executive control functioning, and explore the long-term impact of these factors in mono- and bilingual individuals.

### Source Localization: Proposal of a Cascading Neurophysiological Model of Executive Control Processes in Bilinguals

**FIGURE 6:**
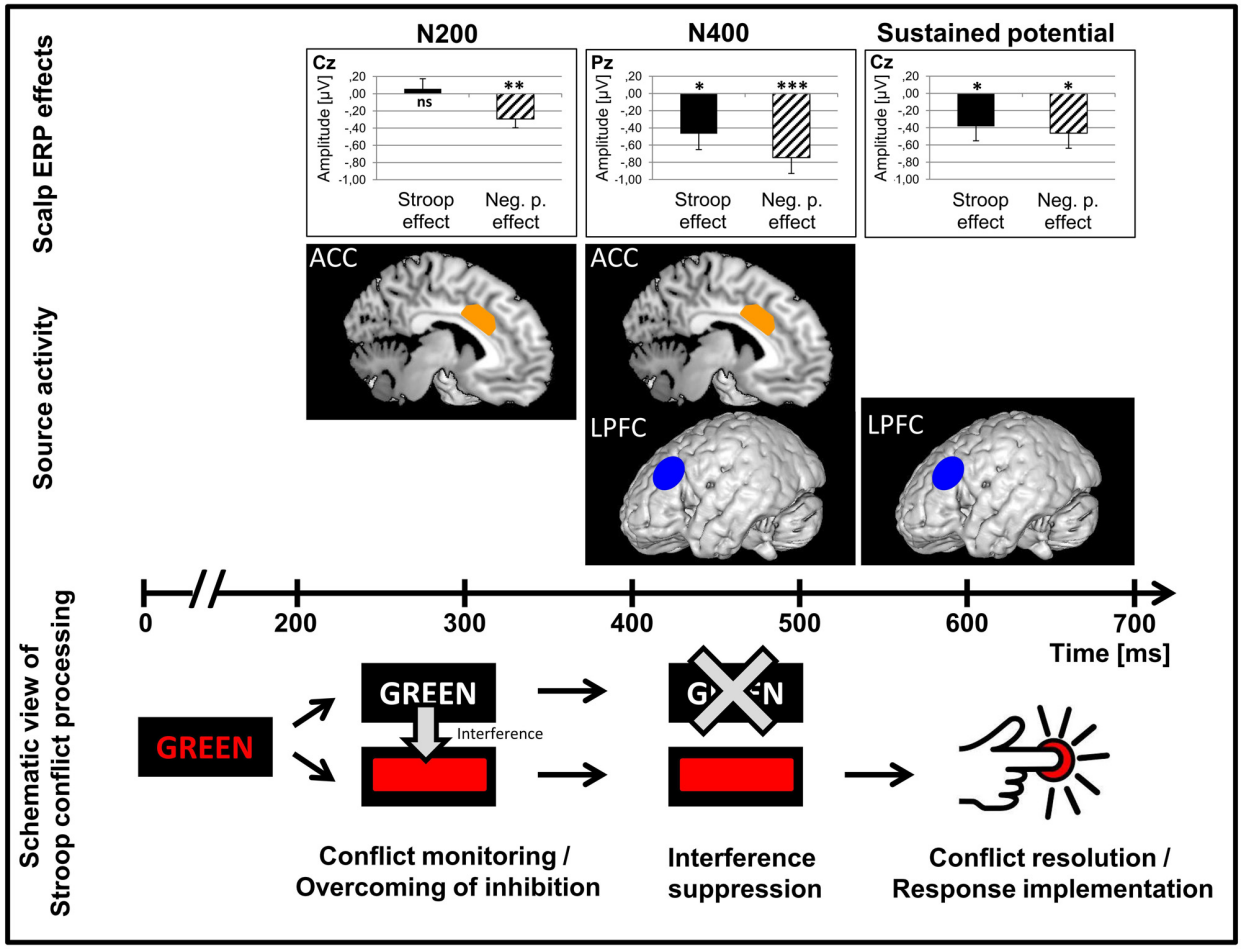
**Schematized view of control processes and the neuronal underpinnings in a Stroop task**. Scalp ERP effect sizes (Stroop effect, Negative priming effect) for each of the three ERP components (N200, N400, late sustained negative-going potential) and the main underlying neuronal sources for the scalp ERP effects are plotted. A schematized view of the time course of Stroop conflict processing is displayed below. Data are collapsed over Language group (*n* = 44). ACC, anterior cingulate cortex; LPFC, left prefrontal cortex. *p < 0.05; **p < 0.01; ***p < 0.001. Image credit for the schematized image of pressing a button: Download Clipart (2013).

As already found in previous studies (Folstein and Van Petten, 2008; Hanslmayr et al., 2008; Bruchmann et al., 2010; among others), we found that the ACC as well as the PFC were main neuronal generators of the N200 and N400 Stroop and Negative priming effects in the present study (**Figures 4** and **6**). Moreover, the present data allow us to precise the time course of these main generators. While in the N200 and the N400 time windows, the ACC showed high activation and was a main neuronal generator for scalp interference effects, its activation did not play a major role in the late sustained negative-going potential time window (for similar findings on transient ACC activation in conflict trials, see Carter et al., 2000). The PFC, on the contrary, was a main neuronal generator for scalp interference effects in the N400 and late sustained negative-going potential time windows. This pattern of ACC and PFC activation was mainly driven by the bilingual group. Thus, our data suggest that the ACC may play a major role in initiating transient control as necessary when conflict has been detected while the PFC would be more active for implementing the control when the need has been detected (i.e., applying inhibition and conflict resolution), which is in line with previous findings and theoretical accounts (Dreher and Berman, 2002; Botvinick, 2007; Carter and Van Veen, 2007; Abutalebi and Green, 2008; Shenhav et al., 2013). However, it has been shown that there are functional subdivisions of the ACC that behave differently according to task demands and are affected differently by task practice (Leung et al., 2000; Milham and Banich, 2005).

Concerning the group differences observed in source activation but not in behavioral data in the present study, note that a similar pattern of results has been reported in a previous MEG study (Bialystok et al., 2005). In this MEG study using a Simon task, Bialystok et al. (2005) found that underlying neuronal processes in the Simon task were different for bilinguals compared to monolinguals even if the groups did not differ in response speed. Bialystok et al. (2005) found that the language group differences did not only consist in the differential intensity of activation of the areas involved in performing the Simon task but even more so in the pattern of areas that were involved. Beyond differences in other areas, in bilinguals as well as monolinguals the incongruency effect was reflected by activation in the left PFC and ACC (among others) but this activation was stronger in bi- than in monolinguals. It is particularly interesting that our data are compatible with these observations since we were using a different task, the Stroop task, which is however, comparable to the Simon task in that both involve conflict processing and are thought to necessitate interference suppression amongst the executive functions. Our results are in line with the Bialystok et al.’s (2005) in two ways, both of which concern especially the differential involvement of the ACC in the two groups: (1) the differences in the pattern of control region involvement in bilinguals and monolinguals in performing the Stroop task, and (2) the more salient difference in source activation between the incongruent and the congruent condition in bilinguals as compared to monolinguals, a group difference that is however, not reflected at the behavioral level. These findings indicate that multiple language use impacts the activation in the neuronal basis of domain-general control processes not only quantitatively in potentially leading to more efficient control but also seems to qualitatively modulate the activation of the control network. The absence of a behavioral bilingual advantage in the present study may be due to the fact that behavioral measures constitute the end-product of a combination of subprocesses, which could mask some effects that are difficult to be traced because of intrinsic heterogeneity of bilingual participants (for similar findings, see Gathercole et al., 2010; Coderre and van Heuven, 2014; Duñabeitia et al., 2014; however, other studies did find a bilingual advantage in a Stroop task, see Bialystok et al., 2008; Heidlmayr et al., 2014; Yow and Li, 2015; for a review, see Paap and Greenberg, 2013).

Further investigation of the neuronal processes in bilingualism should include fMRI studies to obtain higher spatial accuracy as well as functional connectivity analyses. Indeed, Crinion et al. (2006) suggested that subcortical regions like the left caudate may play a crucial role in monitoring and controlling the language in use. Consequently, the description of the neuronal network supporting executive control processes in language control cannot escape a better understanding of how different cortical (ACC, PFC among others) and subcortical (left caudate) brain areas communicate in monitoring and controlling the language in use.

Summing up, bilinguals seem to benefit from higher efficiency in their neuronal and cognitive processing of control implementation, namely interference suppression and conflict resolution because of their experience in handling two languages on a daily basis. However, there appears to be less of an advantage in conflict monitoring, at least for the type of bilinguals selected and the paradigm used in the present study. Moreover, this advantage of bilingualism was not observed in the Negative priming task. Yet, future research using different neuroimaging techniques should help to give a more detailed account of the current findings in trying to characterize the relation between conflict monitoring and interference suppression and the impact of bilingualism in each of these processes. Identifying the neuronal sources of these processes as well as their connectivity with higher precision would be of greatest interest. Moreover, the requirement to deal with linguistic conflict or complexity is not limited to the case of bilingualism but control processing is also crucial in handling within-language interference, as it has been shown for ambiguity resolution in the domains of semantics (Rodd et al., 2010), and syntax (January et al., 2009), but also for phonology, and phonetics (e.g., tongue twisters, Acheson and Hagoort, 2014). Whether control processing involved in managing between- versus within-language interference is quantitatively and/or qualitatively different is still unclear. Further behavioral and neuroscientific research will be necessary to advance our understanding of the similarities and differences between bilingual and monolingual language control.

## Conclusion

The present findings are partially in line with previous studies demonstrating a bilingual advantage on interference control, and more specifically interference suppression. We were able to show a bilingual advantage in the Stroop task but only in the N400 and the late sustained negative-going potential time windows. Unexpectedly, however, we failed to find a bilingual benefit in the Negative priming task, though considered a more complex task. We proposed that this lack of an effect may be due to the specific task demands of the Negative priming task. Nevertheless, the current results are compatible with the hypothesis that bilingualism enhances efficiency of domain-general cognitive control because the neuronal network of general control and the multiple language control network largely overlap (Abutalebi and Green, 2008). Interestingly, we were able to confirm an activation of ACC and PFC which Dreher and Berman (2002) have already established in an fMRI study using a task-switching paradigm, with the Stroop and the Negative priming paradigm, allowing to test conflict monitoring and interference suppression. One of the innovative contributions of our study is the demonstration that there are differential time courses of the involvement of ACC and PFC in conflict processing. While the ACC showed major activation in early time windows (N200 and N400) but not in the later one (late sustained negative-going potential), the PFC became active in the left hemisphere in the N400 time window and in the late sustained negative-going potential time windows. This chronometric finding adds an important piece to the puzzle of theories of the functional relationship between ACC and PFC postulating that ACC would participate in conflict monitoring and communicate with the lateral PFC that would implement cognitive control (MacDonald et al., 2000; Shenhav et al., 2013; for a schematic overview, see **Figure 6**). Further research, combining fMRI and ERP measures, will be necessary to study with both high temporal and spatial resolution the neurochronometry of the cognitive control network, involving amongst others the ACC and the PFC. Moreover, our results are a valuable contribution to the bilingualism literature in that we were able to show that there are specific control processes that seem to be involved in and improved by multiple language use while this may not be the case for other control processes. However, caution is at order before drawing firm conclusions regarding the relation between multiple language use and efficiency of executive functioning. In the present study, the contradictory findings between tasks challenge the view of a systematic bilingualism advantage. On the contrary, the task differences can be explained by assuming that the efficiency of executive functions could also be improved in monolinguals when the experimental design leads them to expect a need for inhibition, thus encouraging an inhibition strategy. Thus, in future research, it will be relevant to apply a battery of tasks tapping into different cognitive and executive functions while taking into consideration the multidimensional characteristics of bilingualism. Such an approach will improve our understanding of the impact of multiple language use on the plasticity of the cognitive control efficiency.

To sum up, the main contribution of the present study is three-fold: our findings indicate that (1) studying the neurodynamics of conflict processing with high temporal resolution can help us disentangling different sub-processes of conflict processing, (2) cascading models appear to capture essential aspects of the time course of neuronal source activation in conflict processing and (3) bilinguals seem to perform better on specific control processes while performing equally to monolinguals on others. Hence, our findings are a valuable contribution to the executive function literature in general and to new theoretical accounts of neurodynamics of executive control in bilingualism in particular.

## Conflict of Interest Statement

The authors declare that the research was conducted in the absence of any commercial or financial relationships that could be construed as a potential conflict of interest.

## Footnotes

1 In case of more negative average amplitudes in the incongruent or negative priming compared to the congruent condition, effect sizes were calculated by subtracting the values in the incongruent or negative priming condition from the values in the congruent condition.

## Abbreviations

ACC: anterior cingulate cortex
LIFG: left inferior frontal gyrus
LMC: left motor cortex
LMTC: left medio-temporal cortex
LOC: left occipital cortex
LPFC: left prefrontal cortex
RIFG: right inferior frontal gyrus
RMC: right motor cortex
ROC: right occipital cortex
RPFC: right prefrontal cortex.

## References

[B1] Abutalebi J. Green D. W. (2008). Control mechanisms in bilingual language production: neural evidence from language switching studies. *Lang. Cogn. Process.* 23 557–582. 10.1080/01690960801920602

[B2] Acheson D. J. Hagoort P. (2014). Twisting tongues to test for conflict-monitoring in speech production. *Front. Hum. Neurosci.* 8:206 10.3389/fnhum.2014.00206 PMC3997035 24795592

[B3] Antón E. Duñabeitia J. A. Estévez A. Hernández J. A. Castillo A. Fuentes L. J. (2014). Is there a bilingual advantage in the ANT task? Evidence from children. *Front. Psychol.* 5:398 10.3389/fpsyg.2014.00398 PMC4019868 24847298

[B4] Appelbaum L. G. Meyerhoff K. L. Woldorff M. G. (2009). Priming and backward influences in the human brain: processing interactions during the Stroop interference effect. *Cereb. Cortex* 19 2508–2521. 10.1093/cercor/bhp036 19321654 PMC2764508

[B5] Aron A. R. (2007). The neural basis of inhibition in cognitive control. *Neuroscientist* 13 214–228. 10.1177/1073858407299288 17519365

[B6] Baum S. Titone D. (2014). Moving toward a neuroplasticity view of bilingualism, executive control, and aging. *Appl. Psycholinguist.* 35 857–894. 10.1017/S0142716414000174

[B7] Bialystok E. Craik F. I. M. Grady C. Chau W. Ishii R. Gunji A. (2005). Effect of bilingualism on cognitive control in the Simon task: evidence from MEG. *Neuroimage* 24 40–49. 10.1016/j.neuroimage.2004.09.044 15588595

[B8] Bialystok E. Craik F. Luk G. (2008). Cognitive control and lexical access in younger and older bilinguals. *J. Exp. Psychol. Learn. Mem. Cogn.* 34 859–873. 10.1037/0278-7393.34.4.859 18605874

[B9] Bialystok E. DePape A.-M. (2009). Musical expertise, bilingualism, and executive functioning. *J. Exp. Psychol. Hum. Percept. Perform.* 35 565–574. 10.1037/a0012735 19331508

[B10] Blumenfeld H. K. Marian V. (2013). Parallel language activation and cognitive control during spoken word recognition in bilinguals. *J. Cogn. Psychol.* 25 547–567. 10.1080/20445911.2013.812093 PMC3827904 24244842

[B11] Boenke L. T. Ohl F. W. Nikolaev A. R. Lachmann T. Leeuwen C. V. (2009). Different time courses of Stroop and Garner effects in perception — an event-related potentials study. *Neuroimage* 45 1272–1288. 10.1016/j.neuroimage.2009.01.019 19349240

[B12] Botvinick M. M. (2007). Conflict monitoring and decision making: reconciling two perspectives on anterior cingulate function. *Cogn. Affect. Behav. Neurosci.* 7 356–366. 10.3758/CABN.7.4.356 18189009

[B13] Bruchmann M. Herper K. Konrad C. Pantev C. Huster R. J. (2010). Individualized EEG source reconstruction of Stroop interference with masked color words. *Neuroimage* 49 1800–1809. 10.1016/j.neuroimage.2009.09.032 19781651

[B14] Carter C. S. Macdonald A. M. Botvinick M. Ross L. L. Stenger V. A. Noll D. (2000). Parsing executive processes: strategic vs. evaluative functions of the anterior cingulate cortex. *Proc. Natl. Acad. Sci. U.S.A.* 97 1944–1948. 10.1073/pnas.97.4.1944 10677559 PMC26541

[B15] Carter C. S. Van Veen V. (2007). Anterior cingulate cortex and conflict detection: an update of theory and data. *Cogn. Affect. Behav. Neurosci.* 7 367–379. 10.3758/CABN.7.4.367 18189010

[B16] Coderre E. L. Conklin K. van Heuven W. J. B. (2011). Electrophysiological measures of conflict detection and resolution in the Stroop task. *Brain Res.* 1413 51–59. 10.1016/j.brainres.2011.07.017 21840503

[B17] Coderre E. L. van Heuven W. J. B. (2014). Electrophysiological explorations of the bilingual advantage: evidence from a Stroop task. *PLoS ONE* 9:e103424 10.1371/journal.pone.0103424 PMC4113364 25068723

[B18] Costa A. Hernández M. Costa-Faidella J. Sebastián-Gallés N. (2009). On the bilingual advantage in conflict processing: now you see it, now you don’t. *Cognition* 113 135–149. 10.1016/j.cognition.2009.08.001 19729156

[B19] Crinion J. Turner R. Grogan A. Hanakawa T. Noppeney U. Devlin J. T. (2006). Language control in the bilingual brain. *Science* 312 1537–1540. 10.1126/science.1127761 16763154

[B20] Dahlin E. Nyberg L. Bäckman L. Neely A. S. (2008). Plasticity of executive functioning in young and older adults: immediate training gains, transfer, and long-term maintenance. *Psychol. Aging* 23 720–730. 10.1037/a0014296 19140643

[B21] Dalrymple-Alford E. C. Budayr B. (1966). Examination of some aspects of the Stroop color-word test. *Percept. Mot. Skills* 23 1211–1214. 10.2466/pms.1966.23.3f.1211 5972923

[B22] Diamond A. Lee K. (2011). Interventions shown to aid executive function development in children 4 to 12 years old. *Science* 333 959–964. 10.1126/science.1204529 21852486 PMC3159917

[B23] Download Clipart. (2013). *Press-Button-Design [png].* Available at: http://www.downloadclipart.net/browse/19591/press-button-clipart [accessed October 16 2014].

[B24] Dreher J.-C. Berman K. F. (2002). Fractionating the neural substrate of cognitive control processes. *Proc. Natl. Acad. Sci. U.S.A.* 99 14595–14600. 10.1073/pnas.222193299 12391312 PMC137928

[B25] Duñabeitia J. A. Hernández J. A. Antón E. Macizo P. Estévez A. Fuentes L. J. (2014). The inhibitory advantage in bilingual children revisited: myth or reality? *Exp. Psychol.* 61 234–251. 10.1027/1618-3169/a000243 24217139

[B26] Dye M. W. G. Green C. S. Bavelier D. (2009). The development of attention skills in action video game players. *Neuropsychologia* 47 1780–1789. 10.1016/j.neuropsychologia.2009.02.002 19428410 PMC2680769

[B27] Folstein J. R. Van Petten C. (2008). Influence of cognitive control and mismatch on the N2 component of the ERP: a review. *Psychophysiology* 45 152–170. 10.1111/j.1469-8986.2007.00602.x 17850238 PMC2365910

[B28] Gathercole V. C. M. Thomas E. M. Jones L. Guasch N. V. Young N. Hughes E. K. (2010). Cognitive effects of bilingualism: digging deeper for the contributions of language dominance, linguistic knowledge, socio-economic status and cognitive abilities. *Int. J. Biling. Educ. Biling.* 13 617–664. 10.1080/13670050.2010.488289

[B29] Gathercole V. C. M. Thomas E. M. Kennedy I. Prys C. Young N. Viñas Guasch N. (2014). Does language dominance affect cognitive performance in bilinguals? Lifespan evidence from preschoolers through older adults on card sorting, Simon, and metalinguistic tasks. *Front. Psychol.* 5:11 10.3389/fpsyg.2014.00011 PMC3914397 24550853

[B30] Gollan T. H. Montoya R. I. Fennema-Notestine C. Morris S. K. (2005). Bilingualism affects picture naming but not picture classification. *Mem. Cognit.* 33 1220–1234. 10.3758/BF03193224 16532855

[B31] Grant A. Dennis N. A. Li P. (2014). Cognitive control, cognitive reserve, and memory in the aging bilingual brain. *Front. Psychol.* 5:1401 10.3389/fpsyg.2014.01401 PMC4253532 25520695

[B32] Green D. W. Abutalebi J. (2013). Language control in bilinguals: the adaptive control hypothesis. *J. Cogn. Psychol.* 25 515–530. 10.1080/20445911.2013.796377 PMC4095950 25077013

[B33] Greenhouse S. Geisser S. (1959). On methods in the analysis of profile data. *Psychometrica* 24 95–112. 10.1007/BF02289823

[B34] Hanslmayr S. Pastötter B. Bäuml K.-H. Gruber S. Wimber M. Klimesch W. (2008). The electrophysiological dynamics of interference during the Stroop task. *J. Cogn. Neurosci.* 20 215–225. 10.1162/jocn.2008.20020 18275330

[B35] Heidlmayr K. Moutier S. Hemforth B. Courtin C. Tanzmeister R. Isel F. (2014). Successive bilingualism and executive functions: the effect of second language use on inhibitory control in a behavioural Stroop Colour Word task. *Biling. Lang. Cogn.* 17 630–645. 10.1017/S1366728913000539

[B36] Hernández M. Costa A. Fuentes L. J. Vivas A. B. Sebastián-Gallés N. (2010). The impact of bilingualism on the executive control and orienting networks of attention. *Biling. Lang. Cogn.* 13 315–325. 10.1017/S1366728909990010

[B37] Hilchey M. D. Klein R. M. (2011). Are there bilingual advantages on nonlinguistic interference tasks? Implications for the plasticity of executive control processes. *Psychon. Bull. Rev.* 18 625–658. 10.3758/s13423-011-0116-7 21674283

[B38] Hoshino N. Thierry G. (2011). Language selection in bilingual word production: electrophysiological evidence for cross-language competition. *Brain Res.* 1371 100–109. 10.1016/j.brainres.2010.11.053 21108940

[B39] Isel F. Aparicio X. Heidlmayr K. Lemoine C. Doré-Mazars K. (2012). Does multilingual code switching impact eye-movement control? Evidence from pro- and anti-saccade tasks. *Poster Presentation Presented at the GDR Vision*, Marseille.

[B40] January D. Trueswell J. C. Thompson-Schill S. L. (2009). Co-localization of Stroop and syntactic ambiguity resolution in Broca’s area: implications for the neural basis of sentence processing. *J. Cogn. Neurosci.* 21 2434–2444. 10.1162/jocn.2008.21179 19199402 PMC2762484

[B41] Keppel G. (1991). *Design and Analysis*, 3rd Edn Englewood Cliffs, NJ: Prentice-Hall.

[B42] Kousaie S. Phillips N. A. (2012). Conflict monitoring and resolution: are two languages better than one? Evidence from reaction time and event-related brain potentials. *Brain Res.* 1446 71–90. 10.1016/j.brainres.2012.01.052 22356886

[B43] Kovacs A. M. Mehler J. (2009). Flexible learning of multiple speech structures in bilingual infants. *Science* 325 611–612. 10.1126/science.1173947 19589962

[B44] Kroll J. F. Bialystok E. (2013). Understanding the consequences of bilingualism for language processing and cognition. *J. Cogn. Psychol.* 25 497–514. 10.1080/20445911.2013.799170 PMC3820916 24223260

[B45] Kuipers J.-R. Thierry G. (2013). ERP-pupil size correlations reveal how bilingualism enhances cognitive flexibility. *Cortex* 49 2853–2860. 10.1016/j.cortex.2013.01.012 23453792

[B46] Laird A. R. McMillan K. M. Lancaster J. L. Kochunov P. Turkeltaub P. E. Pardo J. V. (2005). A comparison of label-based review and ALE meta-analysis in the Stroop task. *Hum. Brain Mapp.* 25 6–21. 10.1002/hbm.20129 15846823 PMC6871676

[B47] Leung H.-C. Skudlarski P. Gatenby J. C. Peterson B. S. Gore J. C. (2000). An event-related functional MRI study of the Stroop color word interference task. *Cereb. Cortex* 10 552–560. 10.1093/cercor/10.6.552 10859133

[B48] Li P. Legault J. Litcofsky K. A. (2014). Neuroplasticity as a function of second language learning: anatomical changes in the human brain. *Cortex* 58 301–324. 10.1016/j.cortex.2014.05.001 24996640

[B49] Liotti M. Woldorff M. G. Perez R. III Mayberg H. S. (2000). An ERP study of the temporal course of the Stroop color-word interference effect. *Neuropsychologia* 38 701–711. 10.1016/S0028-3932(99)00106-2 10689046

[B50] Liu C. Yao R. Wang Z. Zhou R. (2014). N450 as a candidate neural marker for interference control deficits in children with learning disabilities. *Int. J. Psychophysiol.* 93 70–77. 10.1016/j.ijpsycho.2014.05.007 24858538

[B51] MacDonald A. W. Cohen J. D. Stenger V. A. Carter C. S. (2000). Dissociating the role of the dorsolateral prefrontal and anterior cingulate cortex in cognitive control. *Science* 288 1835–1838. 10.1126/science.288.5472.1835 10846167

[B52] MacLeod C. M. MacDonald P. A. (2000). Interdimensional interference in the Stroop effect: uncovering the cognitive and neural anatomy of attention. *Trends Cogn. Sci.* 4 383–391. 10.1016/S1364-6613(00)01530-8 11025281

[B53] Markela-Lerenc J. Ille N. Kaiser S. Fiedler P. Mundt C. Weisbrod M. (2003). Prefrontal-cingulate activation during executive control: which comes first? *Cogn. Brain Res.* 18 278–287. 10.1016/j.cogbrainres.2003.10.013 14741314

[B54] Marzecová A. Bukowski M. Correa Á. Boros M. Lupiáñez J. Wodniecka Z. (2013). Tracing the bilingual advantage in cognitive control: the role of flexibility in temporal preparation and category switching. *J. Cogn. Psychol.* 25 586–604. 10.1080/20445911.2013.809348

[B55] Milham M. P. Banich M. T. (2005). Anterior cingulate cortex: an fMRI analysis of conflict specificity and functional differentiation. *Hum. Brain Mapp.* 25 328–335. 10.1002/hbm.20110 15834861 PMC6871683

[B56] Miyake A. Friedman N. P. Emerson M. J. Witzki A. H. Howerter A. Wager T. D. (2000). The unity and diversity of executive functions and their contributions to complex “Frontal Lobe” tasks: a latent variable analysis. *Cogn. Psychol.* 41 49–100. 10.1006/cogp.1999.0734 10945922

[B57] Morton J. B. Harper S. N. (2007). What did Simon say? Revisiting the bilingual advantage. *Dev. Sci.* 10 719–726. 10.1111/j.1467-7687.2007.00623.x 17973787

[B58] Naylor L. J. Stanley E. M. Wicha N. Y. Y. (2012). Cognitive and electrophysiological correlates of the bilingual Stroop effect. *Front. Psychol.* 3:81 10.3389/fpsyg.2012.00081 PMC3317261 22485099

[B59] Paap K. R. Greenberg Z. I. (2013). There is no coherent evidence for a bilingual advantage in executive processing. *Cogn. Psychol.* 66 232–258. 10.1016/j.cogpsych.2012.12.002 23370226

[B60] Peterson B. S. Kane M. J. Alexander G. M. Lacadie C. Skudlarski P. Leung H.-C. (2002). An event-related functional MRI study comparing interference effects in the Simon and Stroop tasks. *Cogn. Brain Res.* 13 427–440. 10.1016/S0926-6410(02)00054-X 11919006

[B61] Prior A. Macwhinney B. (2009). A bilingual advantage in task switching. *Biling. Lang. Cogn.* 13 253–262. 10.1017/S1366728909990526 PMC9724810 36479004

[B62] Rodd J. M. Johnsrude I. S. Davis M. H. (2010). The role of domain-general frontal systems in language comprehension: evidence from dual-task interference and semantic ambiguity. *Brain Lang.* 115 182–188. 10.1016/j.bandl.2010.07.005 20709385

[B63] Shenhav A. Botvinick M. M. Cohen J. D. (2013). The expected value of control: an integrative theory of anterior cingulate cortex function. *Neuron* 79 217–240. 10.1016/j.neuron.2013.07.007 23889930 PMC3767969

[B64] Stroop J. R. (1935). Studies of interference in serial verbal reactions. *J. Exp. Psychol.* 18 643–662. 10.1037/h0054651

[B65] Valian V. (2015). Bilingualism and cognition. *Biling. Lang. Cogn.* 18 3–24. 10.1017/S1366728914000522

[B66] Van Heuven W. J. B. Dijkstra T. Grainger J. (1998). Orthographic neighborhood effects in bilingual word recognition. *J. Mem. Lang.* 39 458–483. 10.1006/jmla.1998.2584

[B67] West R. (2003). Neural correlates of cognitive control and conflict detection in the Stroop and digit-location tasks. *Neuropsychologia* 41 1122–1135. 10.1016/S0028-3932(02)00297-X 12667546

[B68] Yow W. Q. Li X. (2015). Balanced bilingualism and early age of second language acquisition as the underlying mechanisms of a bilingual executive control advantage: why variations in bilingual experiences matter. *Front. Psychol.* 6:164 10.3389/fpsyg.2015.00164 PMC4341428 25767451

